# Intramammary antimicrobial sales in Ireland: a 2020 descriptive update

**DOI:** 10.1186/s13620-022-00213-w

**Published:** 2022-03-26

**Authors:** Catherine I. McAloon, Finola McCoy, Simon J. More

**Affiliations:** 1 UCD School of Veterinary Medicine, University College Dublin, Belfield, Dublin, D04 V1W8 Ireland; 2 Animal Health Ireland, 4-5 The Archways, Carrick on Shannon, Co., Leitrim N41 WN27 Ireland

**Keywords:** Antimicrobial resistance, Intramammary antimicrobial, Sales data

## Abstract

Intramammary (IM) antimicrobial sales data are currently the only feasible means to gain broad insights into on-farm usage of antimicrobials (AMs) relevant to mastitis control within the Irish dairy industry. The aim of this study was to update earlier work describing sales data of intramammary antimicrobial usage in the Irish dairy industry in 2020. Previously reported data from 2013 to 2019 is included for reference and 2020 sales data is reported using similar methodology to previously published work in this area. Data on IM AMs sold in Ireland during 2013-2020 were obtained from two sources, believed to represent 99% of all sales of IM AMs in Ireland, and analyses were undertaken to evaluate patterns in IM AM sales. We report an increase in overall sales of both lactating cow (LC) and dry cow (DC) IM AMs. We observed a large increase in the use of DC IM AMs, from 0.95 to 1.13 defined course dose (DCDvet) per cow per year in 2019 and 2020 respectively, as well as evidence of ongoing usage of highest priority critically important AMs, as defined by the World Health Organization. There was also a slight increase in LC use of IM AMs, from 0.43 to 0.44 defined course dose (DCDvet) per cow per year. We believe that our results provide an accurate reflection of IM sales in Ireland in 2020. In common with any study of this type, caution is needed when interpreting national IM AM sales data, noting the potential discrepancies between AM sales and on-farm usage. Nonetheless, the sales pattern described here, most importantly the increased use of DC products and ongoing and increasing use of HP CIA products in both DC and LC therapy raise significant concerns for the Irish dairy industry. This study provides an evidence base to inform current policy discussions, particularly in the context of the new Veterinary Medicines Regulation (Regulation (EU) 2019/6), which comes into force on 28 January 2022.

## Introduction

In a number of European countries, including Belgium [1], Denmark [2] and the Netherlands [3], the objective measurement of on-farm antimicrobial (AM) usage has proved central to progress towards improved AM stewardship in farm animal production [4] (More, 2020). Further, the collection of data on the sale and use of AMs at prescriber level is a key requirement for member states within the new Veterinary Medicine Regulations (Regulation (EU) 2019/6) [5].

In Ireland, a national prescribing database for all prescription only medicines including antimicrobials (the National Veterinary Prescribing System, NVPS) is under development. For this reason, intramammary (IM) AM sales data are currently the only feasible means to gain broad insights into on-farm usage of AMs relevant to mastitis control within the Irish dairy industry. Published information is available for the period 2003-19 [6–8], with key themes including near-universal blanket dry cow therapy across the national herd until very recently, a progressive fall in in-lactation therapy since 2013 (to a defined course dose (DCD)/cow of 0.43 in 2019), and ongoing concerns about both the use and trend in use of highest priority critically important AMs (HP CIA) in IM products, both with in-lactation and dry-cow therapies.

Ongoing updates of these sales data are important, providing insights into estimated usage to inform decision-making, both by industry and government. This information is also central to discussions within CellCheck, the national mastitis control programme managed by Animal Health Ireland [9]. An update is particularly important at this time, to inform national discussion and action in preparation for the new Veterinary Medicines Regulation, which come into force from 28 January 2022.

Therefore, the aim of this study was to update the earlier work of More et al. [7] and McAloon et al. [8] as a descriptive update of IM AM usage in the Irish dairy industry to include 2020 sales data. The data from 2013 to 2019 has already been reported and is included for reference purposes, reported comparisons are between 2019 and 2020 only.

## Materials and methods

The materials and methods used were as described by McAloon et al. (2021) [8]. Briefly, data on IM AMs sold in Ireland during 2013-2020 were obtained from two sources. Sales data for 2013-2020 were obtained from Kynetec (Newbury, Berkshire, UK), which is an international market research company that gathers data on all IM AM sales conducted through the main drug wholesalers. In addition, sales data for 2011-2020 was provided by 1 manufacturer, sales data for 2013-2020 from another 2 distributors of IM AMs, as well as data supplied by 2 other distributors who began selling IM AMs in 2020 only, all of whose sales data are not supplied directly to Kynetec. These datasets, which are believed to represent all relevant sources of IM AMs sold in Ireland during this period, were subsequently reconciled to avoid any data duplication. The population of interest is all dairy cows in Ireland, and dairy cow numbers were obtained from Eurostat [10]. The WHO (World Health Organisation) classification of AMs reflects the importance of different AM groups for human medicine [11]; either very important, critically important (CIA) or highest priority critically important AMs (HP CIAs). This classification system was used to analyse the type of IM AM products sold.

The numbers of IM AM tubes sold each year, by product type (in-lactation (LC) therapy, dry cow (DC) therapy), and by WHO classification were calculated. On-farm AM usage was estimated using the technical units daily defined dose (DDDvet) and DCDvet per cow per year [7]. In these calculations, and also to calculate the number of cows eligible for dry cow treatment, mean intercalving intervals of 391, 391, 391, 388, 390, 387, 390 and 387 days during 2013 to 2020 respectively were used, based on data from the Irish Cattle Breeding Federation (ICBF) for herds with greater than 30 calvings annually [12], the mean length of the dry period was assumed to be 60 days, and a mean annual replacement rate of 21%, for the years 2003 to 2015, and 22, 21, 21 and 20% for 2016-2020 respectively was used [12]. The number of cows eligible for DC therapy was calculated each year using the number of dairy cows in the country x (1- annual replacement rate) × 365/mean intercalving interval, using the ICBF stated figures. Nulliparous heifers or cows at the end of their final lactation were not assumed to be eligible for DC therapy. This same calculation of the number of cows eligible for dry cow therapy was used in the previous work on IM AM sales in Ireland. It includes 2 parts as firstly to assume only cows at the end of the lactation receive dry cow therapy but not cows that are culled and in addition the second part of the calculation was used to account for a change in median calving date year on year, accounting for the year changes in intercalving interval. More recently the median calving date is more stable and hence in this publication we have used a new formula to calculate the number of cows eligible for dry cow therapy; that is the total number of dairy cows multiplied by 1 minus the replacement rate, this assumes any cow at the end of lactation (except the number to be culled) are eligible for dry cow therapy and no longer includes any account of changes in median calving date. We report the DCDvet per cow per year for dry cows using both calculations (to allow for comparisons with previous work) of the number of cows eligible for dry cow therapy. In these calculations, the sales data were assumed to represent 99% of all IM sales This assumption was determined following discussion with data providers, based on all verified distribution routes for IM AMs into Ireland during these periods. Data management and analysis was conducted in Microsoft Excel (Microsoft Corp., Redmond, WA).

## Results and discussion

**Fig. 1:**
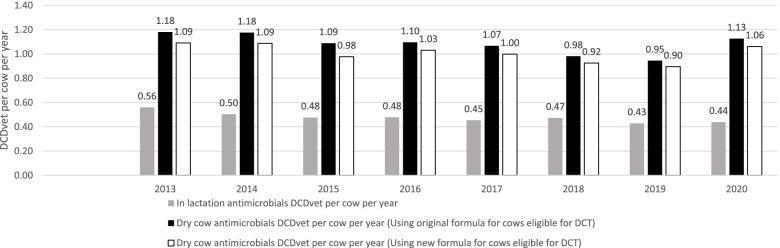
Estimated on-farm intramammary antimicrobial usage for in-lactation and dry cow therapy in Ireland during 2013 to 2020, expressed as defined course dose (DCDvet). These data are based on sales data collated by Kynetec and 5 other individual suppliers, which is assumed to represent 99% of sales data from 2013 onwards. The black bars represent the DCDvet per cow per year for dry cow therapy using the original formula to estimate the number of cows at risk of dry cow therapy. The white bars represent DCDvet per cow per year for dry cow therapy using a new simplified version of the formula to estimate the number of cows at risk of dry cow therapy

**Fig. 2:**
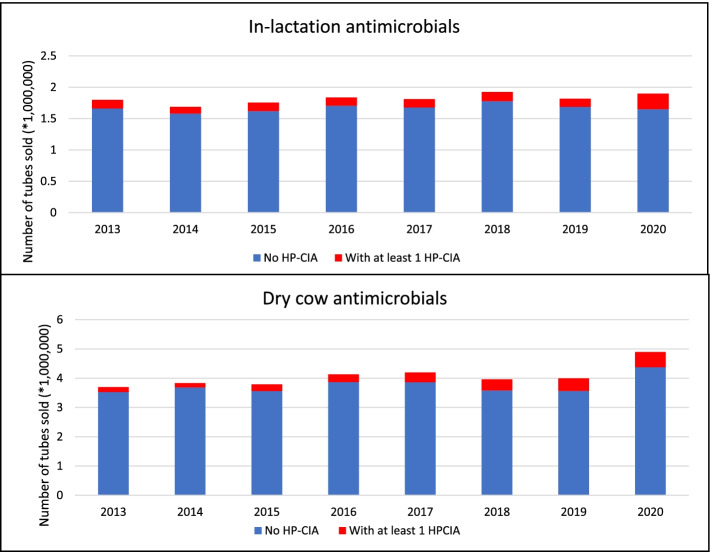
Number of intramammary antimicrobial tubes sold annually in Ireland during 2013-2020, for in-lactation (top) and dry cow (bottom) therapy. The graphs present the number of tubes sold containing none or at least one highest priority critically important antimicrobial (HP-CIA). These data are based on sales data collated by Kynetec and 5 other individual suppliers, which is assumed to represent 99% of sales data

We report an increase in overall sales of both LC and DC IM AMs. Cow-adjusted figures report a slight increase in LC use of IM AMs from 1.29 to 1.32 DDDvet per cow per year or an increase from 0.43 to 0.44 DCDvet per cow per year (Fig. 1) between 2019 and 2020. We also report a large increase in the use of DC IM AMs, with an increase in DCDvet per cow per year from 0.95 to 1.13 DCDvet per cow per year between 2019 and 2020 (or between 0.90 and 1.06 DCDvet per cow per year using the new formula for calculation in the number of cows eligible for dry cow treatment) (Fig. 1). In addition, the number of IM AMs sold containing at least 1 HP CIA increased, more substantially among LC therapy with an increase from 7 to 13% of in-lactation AMs sold containing at least 1 HP CIA between 2019 and 2020 (Fig. 2). Further, based on kg of active substance, there was a large increase in both the DC and LC use of fourth generation cephalosporin specifically. These results are worrying and at odds with national intentions, with regard to DC AM coverage (specifically, a shift from blanket to selective DC therapy), as well as the ongoing use of HP CIA, specifically fourth generation cephalosporins in mastitis treatment and control. Guidelines on the use of HP CIA in veterinary medicine have been developed by DAFM as an output of Ireland’s National Action Plan on Antimicrobial Resistance (iNAP). This advice, published in September 2020, states that use of HP CIA should not be prescribed and/or administered by a veterinarian until results of culture and susceptibility testing are received indicating there is no effective alternative [13].

In common with any study of this type, caution is needed when interpreting national IM AM sales data, noting the potential discrepancies between AM sales and on-farm AM usage [7, 8]. We note that there is the potential for product to be sold and not used, for example due to product stockpiling and/or expiry. Conversely, there is also the potential for leakage of IM AM products into off label use in heifers, suckler cows or indeed for the treatment of pink eye or other conditions for all classes of cattle. A key feature of our work has been the consistency over time in the data sources that have been used, in the methods used to analyse IM AM sales (including the underlying assumptions), and in the interpretation of study results. For this reason, it is the patterns in sales that are of particular importance. Given the observed – and unexpected – increase in IM AM sales in 2020, particularly of DC products, we re-considered the potential for quality issues at each stage of the study. To our knowledge, we have captured all of the sources of IM AM product sold in Ireland. Further, at our request, the data were cross-checked and re-validated by each of the data providers. In doing so, we were able to identify problems with supply of certain products in the previous 18 months due to lack of availability, which may have been a factor in the increase in sales in HP-CIA products, due to a lack of alternatives. We believe that our results provide an accurate reflection of IM sales in Ireland in 2020.

The sales figures described here, most importantly the increased use of DC products and ongoing use of HP CIA products in both LC and DC therapy raise significant concerns for the Irish dairy industry. Regulation 2019/6 frames a major change in how DC therapy is used, and in the importance of preserving HP CIA products, such as fourth generation cephalosporins which are classed by the European Medicines Agency as category B products and should be restricted in animal use unless absolutely necessary [14]. Notwithstanding the above-mentioned challenges associated with potential discrepancies between IM AM sales and usage, as well as the potential for supply shortages to affect product choice, this study provides an evidence base to inform current policy discussions. Major changes will be needed in prescribing practices and for industry education and collaboration to deliver the Veterinary Medicines Regulation safely and effectively. International experience, including from Denmark and the Netherlands [2, 3], has demonstrated the central role played by farm- and prescriber-level benchmarking in the substantial and ongoing reduction in AM usage, both nationally and in specific sectors. Benchmarking would likely achieve a similar impact in Ireland; indeed, it may not be possible to achieve and sustain similar progress towards improved AM stewardship in its absence.

## Data Availability

Raw data is not available due to commercial sensitivity of the data involved.
